# Burdens and resources of Austrian clinical psychologists: results of a qualitative study two years into the COVID-19 pandemic

**DOI:** 10.1186/s40359-024-01714-9

**Published:** 2024-04-13

**Authors:** Andrea Jesser, Agnes Steinböck, Barbara Pammer, Tiam Ghorab, Magdalena Weber, Yvonne Schaffler, Thomas Probst, Anna Felnhofer, Oswald D. Kothgassner, Christoph Pieh, Elke Humer

**Affiliations:** 1 Department for Psychosomatic Medicine and Psychotherapy, University for Continuing Education Krems, Krems, 3500 Austria; 2 Faculty of Psychotherapy Science, Sigmund Freud University Vienna, Vienna, 1020 Austria; 3 Clinical Psychologist and Psychotherapist, Graz, Austria; 4 Division of Psychotherapy, Department of Psychology, Paris Lodron University of Salzburg, Salzburg, 5020 Austria; 5 Department of Pediatrics and Adolescent Medicine, Division of Pediatric Pulmonology, Allergology and Endocrinology, Medical University of Vienna, Vienna, Austria; 6 Department of Child and Adolescent Psychiatry, Comprehensive Center for Pediatrics, Medical University of Vienna, Vienna, Austria

**Keywords:** Clinical psychologists, Pandemic, Resources, Burdens

## Abstract

**Background:**

The COVID-19 pandemic increased the mental health burden in the general population, enhancing the demands placed on mental healthcare professionals.

**Methods:**

This study aimed to assess the burdens and resources of clinical psychologists that emerged since the beginning of the pandemic. *N* = 172 Austrian clinical psychologists participated in a cross-sectional online survey between April and May 2022. The burdens and the sources of support that emerged during the pandemic were analyzed using qualitative content analysis.

**Results:**

Mental health-related issues were identified as the greatest burden, followed by work-related themes and restrictions imposed by the government to combat the spreading of the virus. The most important resources mentioned by the clinical psychologists were social contacts and recreational activities. Practising mindfulness and focusing on inner processes and work-related aspects were further important resources mentioned.

**Conclusion:**

Overall, it seems that clinical psychologists have a high awareness of mental health-related problems related to the pandemic and use adaptive coping strategies to deal with them.

## Background

Various studies have shown that the COVID-19 pandemic had a wide range of effects on society. In addition to the disease itself and the fear of infection, the accompanying measures, such as lockdowns and physical distancing, had a major impact on our lives, particularly our physical and mental health [1–4].

The government in Austria imposed the first lockdown on March 16, 2020. The lockdown was accompanied by major restrictions and ended on May 1, 2020. The obligatory COVID-19 lockdown measures entailed a nationwide curfew with restrictions on movement and activities. Exceptions included addressing immediate danger, meeting basic needs, fulfilling work responsibilities (if unable from home), providing care for those in need, and engaging in outdoor activities. As a second COVID-19 wave followed in the fall of 2020, a second lockdown was decreed from November 17 until December 6, 2020, followed by a third lockdown from December 26 until February 7, 2021. Due to the emergence of the SARS-CoV-2 Delta variant, the number of infected people strongly increased again in the autumn of 2021. A further lockdown was imposed from November 22, 2021. For vaccinated people, the lockdown ended on December 11, 2021, and for unvaccinated people on January 31, 2022. On February 5, 2022, a vaccination obligation came into force in Austria, which was repealed on July 29, 2022. Our study was conducted during April and May 2022. At this time, the Omicron variant was dominant in Austria. After daily highs in confirmed COVID-19 cases from January to March 2022, infection rates declined in April 2022. The milder course of the Omicron variant allowed strong relaxation of the containment efforts in spring 2022. At the time of the survey, only a few measures were in place, such as mandate masking in essential shops, hospitals, and nursing homes and the need to prove a low epidemic risk upon entering Austria [5]. However, the existence of compulsory vaccination at the time of the survey and the socio-political discussion about it did not help to ease the general mood among the population [6].

Already at the time of the first lockdown, mental health problems increased in the general population in Austria, with prevalence rates of 21% for clinically relevant symptoms of depression, 19% for anxiety, and 16% for insomnia [7]. Comparable representative population studies from before the pandemic found that 6% of the population scored above the same cut-off for depression and anxiety [8, 9]. Further studies showed that adverse effects persisted beyond the lockdowns [2, 10] and increased even further, reaching a prevalence of 28% for depression in April 2022 [11].

With the rise in mental health problems, the demand for professional psychological support and the number of patients being treated also increased in Austria [12, 13]. Besides psychiatrists, psychotherapists and clinical psychologists are involved in the treatment of mental health problems in Austria. The professional title “psychotherapist” may only be used by persons who have completed training that meets the requirements of the Austrian Federal Ministry of Social Affairs, Health, Care, and Consumer Protection. There is a wide range of established psychotherapy methods, which can be classified into four orientations (psychodynamic, humanistic, systemic, behavioral) [14]. The training qualifies for treatment based on one of the recognised methods. To work as a clinical psychologist, it is necessary to complete in-depth postgraduate training in addition to a degree in psychology and to have practical experience in a health or social service institution. Clinical psychological treatment includes the use of clinical psychological approaches based on the science of psychology, its findings, theories, methods and techniques [15] and therefore goes beyond single methods. This study focuses on the group of clinical psychologists in Austria.

The well-being of mental health professionals is crucial for successful treatment [16]. Therefore, the question arises as to how much they have been burdened. After all, mental health professionals are also affected by the pandemic and are at risk of experiencing adverse mental health outcomes [17].

Previous studies have shown that health professionals in general were particularly challenged during the pandemic. This is reflected in the collected findings of several meta-analyses, which reported anxious and depressive symptomatology, sleep disorders, or burnout in health professionals [18–20]. However, most of these studies refer to the group of physicians and nurses, and there is less empirical evidence on mental healthcare professions, such as clinical psychologists or psychotherapists. Also, existing studies considering these professions are contradictory.

For example, a study on 1,547 psychotherapists in Austria indicated that their stress level was higher during the pandemic than reference values measured in the general German population before the pandemic [21]. Possible stressors included fear of infection in direct patient contact or changes in everyday practice such as switching to remote psychotherapy, working with a mask, dealing with waiting lists due to increased need for psychotherapeutic treatment, or changes in patient's existing symptoms [22, 23]. Similarly, Rosen et al. [24] reported increased burnout among psychotherapists during the COVID-19 pandemic. Since burnout is related to perceived stress in professional counsellors, this finding also points to increased stress levels among psychotherapists [25].

The extent to which mental health professionals feel stressed can be influenced by their coping strategies. It could be shown that avoidant coping strategies (such as denial, distraction, and substance use) were associated with increased stress levels, which predicted lower well-being. On the other hand, active coping (e.g., positive attitude, problem-solving, social support) positively affected well-being and was negatively associated with psychological distress [26–29]. Resources such as physical activity, relaxation at work, mindfulness-based resilience training programs, or practising autogenic training have been recommended in this context [30].

While the studies mentioned above suggested that mental health professionals were particularly burdened by the pandemic, results of a recent survey by Schaffler et al. [31] indicated that Austrian psychotherapists had fewer problems with depressive, anxiety, insomnia, and stress symptoms than the general population. Similarly, Austrian telephone emergency counsellors have been found to experience less stress and better mental well-being compared to a representative sample of the general population [32, 33].

Studies focusing on clinical psychologists are rare and their results also point in different directions. In 2015, a study of 678 UK-based clinical psychologists found that 63% of them reported having experienced mental health problems at some point in their lives, while the lifetime prevalence of diagnosable mental health problems in the general population was considerably lower at 41% [34]. In contrast, an Austrian study of *N* = 172 clinical psychologists revealed a lower prevalence of depression, anxiety, and clinically relevant stress levels in clinical psychologists compared to the Austrian general population in spring 2022 [35]. 12.2% of clinical psychologists exceeded the cut-off scores for clinically relevant depression and anxiety (compared to 24% and 20% in the general population), 43% reported a moderate or high stress level (compared to 64% in the general population). Another study on healthcare workers in Brazil found that clinical psychologists had the lowest scores regarding the psychological impact of the pandemic. Their training and ability to develop adaptive strategies were discussed as protective factors [36].

In sum, empirical data on the impact of the COVID-19 pandemic on mental health professionals are still scarce and contradictory. Although the pandemic challenged health professionals, Austrian clinical psychologists seem less mentally burdened than the general population [35]. However, the underlying reasons remain unclear so far. To better understand stressors and protective factors clinical psychologists faced two years into the pandemic, our study aimed to investigate their self-reported burdens and the resources.

## Methods

### Study design

Between April 11 and May 31, 2022, a cross-sectional internet-based survey was conducted using Research Electronic Data Capture (REDCap) (Vanderbilt University, Nashville, TN, USA) [37]. The survey constituted 49 items in total. Results on the quantitative analyses are presented in our companion paper [35]. The link to the survey was sent via e-mail to clinical psychologists registered in the list of the Austrian Federal Ministry of Social Affairs, Health, Care and Consumer Protection (>11000 clinical psychologists registered in April 2022), given that they provided a valid e-mail address (≈5000 clinical psychologists). Several clinical psychologists were also registered as psychotherapists in the list of the Austrian Federal Ministry of Social Affairs, Health, Care and Consumer protection. As psychotherapists were also invited to participate in a survey on the same topic (results published in our companion papers [31, 38]), the current analyses encompassed only clinical psychologists without additional license as a psychotherapist (≈ 3000 eligible participants).

The study was conducted after approval by the data protection officer and the Ethics Committee of the University for Continuing Education Krems, Austria (Ethical number: EK GZ 11/2021-2024). All participating clinical psychologists gave electronic informed consent to participate and complete the questionnaires. Clinical psychologists received no compensation for their time and effort, and participation was voluntary.

### Measures

#### Sociodemographic and job-related variables

Data on gender, age, and years in the profession (the time since participants were registered in the official list of licensed clinical psychologists) were collected. All participants were further asked about their employment type (private practice, outpatient institution, inpatient institution) and whether they derived all their income from their clinical psychological treatments. They were further asked about the number of patients treated clinically-psychologically on average per week in personal contact, via the Internet, and the telephone. Other job-related variables surveyed were the treated patient group (children and adolescents, adults) and the setting in which treatment was provided (treating individuals, partners, families, or groups).

#### Open-ended questions on perceived burdens and resources

To evaluate the perceived burdens and resources of clinical psychologists during a period of consecutive crises, the following five free-text questions were asked:

1. What currently burdens you the most?
2. How do these burdens currently show themselves?
3. If you look back today at the last two years: What effects of the pandemic on your mental health and well-being have you observed?
4. What helped you to cope with the adverse effects of the pandemic?
5. Have there also been positive effects due to the pandemic?

Both questions and answers were initially formulated in German. As there were no predefined possible answers, the respondents were allowed to describe their own experiences. Responses ranged from single-word answers to whole paragraphs. It was also possible to skip each of the free-text questions.

### Analyses

Sociodemographic data were analyzed descriptively to describe the characteristics of the sample. The data derived from the open-ended questions were analyzed by two coders using conventional qualitative content analysis, followed by quantifying the qualitative categories [39]. After an initial review of the data, it was decided to analyze the answers to questions 1-3 and 4-5 together. Answers to questions 1-3 could be thematically assigned to clinical psychologists’ burdens and responses to questions 4-5 to their resources.

The first coder analyzed the questions regarding the burdens (Questions 1-3), and the second coder addressed the questions concerning the resources (Questions 4-5). For this purpose, both coders first read all the data to familiarize themselves with the material and gain an overview. The responses were then read again word for word. In this process, categories for questions 1-3 and 4-5 were derived inductively, and category definitions, coding rules, and exemplary citations were documented in a codebook. After that, the coders subsumed subcategories with similar content under more abstract categories. This resulted in one category system for questions 1-3 and one for questions 4-5. The created category systems were then discussed with the research team regarding their applicability.

In the next step, the coders coded their respective datasets with their list of categories using the software ATLAS.ti [40]. As respondents were free to mention several aspects per question, assigning more than one category per response was possible. After the coders had coded the entire data set, they read all quotations assigned to one category. During this process, coding errors were corrected, and definitions and coding rules were made more precise. Subsequently, a third coder coded *N*=50 (28.4%) out of *N*=172 cases according to the coding rules, category definitions, and quote examples defined in the codebook to check for intercoder-reliability. The latter was calculated as the ratio of matching codings divided by the number of cases coded by both coders. The criterion for a matching case required both coders to apply the same categories to the case coded. Our approach resulted in an intercoder-reliability coefficient of *r* = 0.88. The mismatching cases were discussed with the research team, and final adaptations were made to the category systems.

## Results

### Study sample characteristics

A total number of *N* = 172 clinical psychologists participated (≈ 6% response rate). The sample comprised only clinical psychologists without additional training in psychotherapy. Study sample characteristics are summarized in Table 1.

**Table 1:** Characteristics of the participating clinical psychologists

**Variable**	
**Gender**	
Female, *N* (%)	158 (91.9)
Male, *N* (%)	14 (8.1)
**Age in years, M (SD)**	**44.9 (7.97)**
**Professional experience in years, M (SD)**	**13.9 (7.72)**
**Number of patients treated per week, M (SD)**	**14.1 (9.36)**
Proportion of patients treated in personal contact, % (SD)	85.2 (20.96)
Proportion of patients treated via the Internet, % (SD)	7.86 (14.72)
Proportion of patients treated via the telephone, % (SD)	6.93 (14.63)
**Form of employment as clinical psychologist**	
Private practice, % (*N*)	128 (74.4)
Outpatient facility, % (*N*)	65 (37.8)
Inpatient facility, %(*N*)	47 (27.3)
**Income**	
Additional income, % (*N*)	41.3 (71)
Only clinical psychology, % (*N*)	58.7 (101)
**Setting**	
Individuals, % (*N*)	99.4 (171)
Couples, % (*N*)	23.3 (40)
Families, % (*N*)	22.7 (39)
Groups, % (*N*)	28.5 (49)
**Patient group**	
Only adults, % (*N*)	32.0 (55)
Only children and adolescents, % (*N*)	12.8 (22)
Children, adolescents, and adults, % (*N*)	55.2 (95)

### Burdens

Within the questions related to burdens (questions 1-3), out of *N*=172, *N*=152 (88.4%) answered at least one question, *N*=144 (83.7%) at least two questions and *N*=133 (77.3%) answered all three questions.

**Fig. 1:**
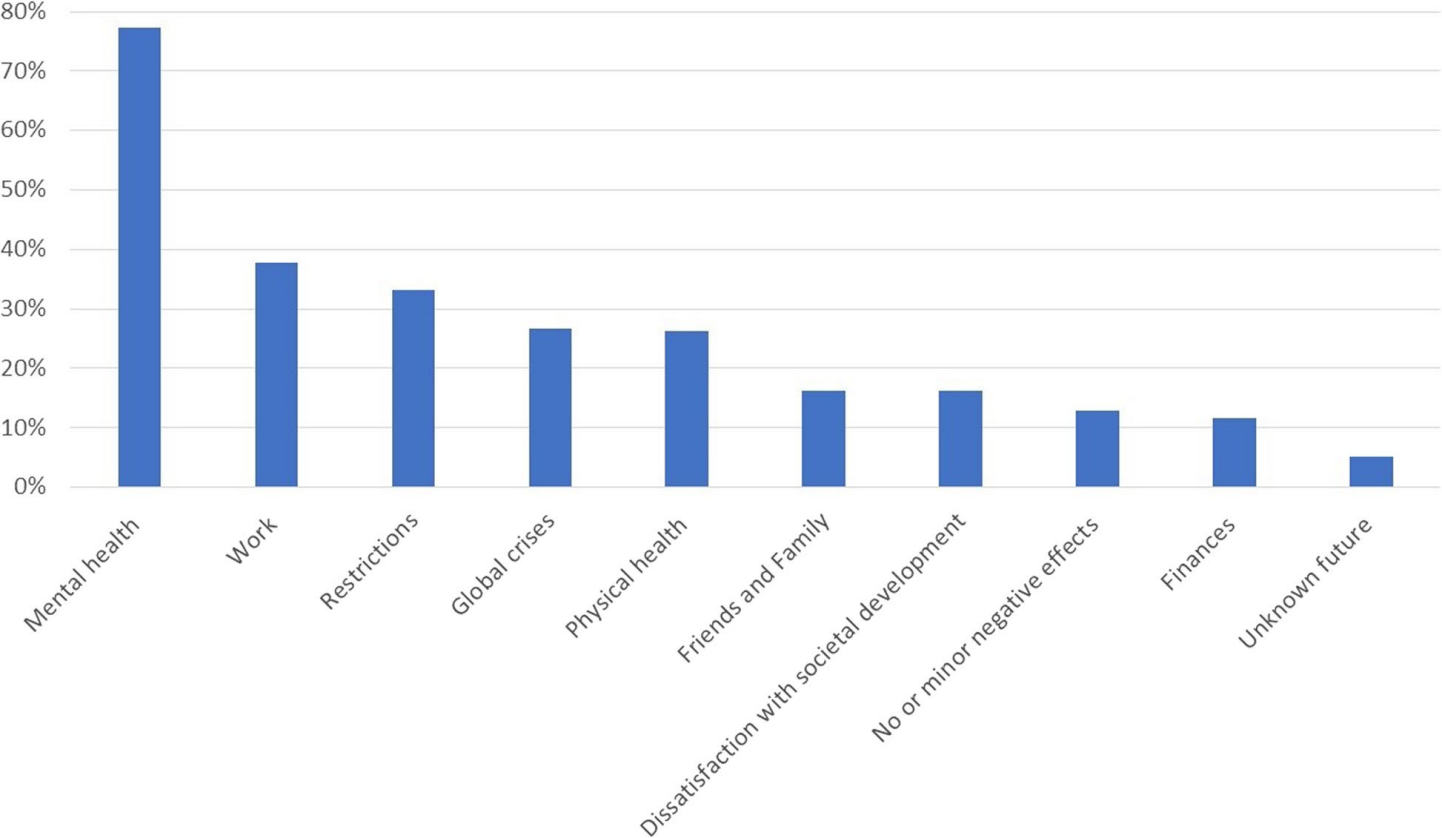
Burdens among clinical psychologists. The percentages of participants reporting one or more burdens in each of the main categories that resulted from the qualitative content analysis of questions 1-3: (Question 1) What burdens you the most at the moment? (Question 2) How do these burdens currently show themselves? (Question 3) If you look back today at the last two years: What effects of the pandemic on your mental health and well-being have you observed?

Qualitative content analysis resulted in 10 categories (Fig. 1) with 21 subcategories. The results are described in detail in Table 2.

**Table 2.** Category system that emerged from the qualitative content analysis of questions 1-3: (Question 1) What burdens you the most at the moment? (Question 2) How do these burdens currently show themselves? (Question 3) If you look back today at the last two years: What effects of the pandemic on your mental health and well-being have you observed?

	***N***	**%**
**Mental health**	**133**	**77.3**
Negative feelings	84	48.8
Symptoms of burnout	63	36.6
Excessive demand	57	33.1
Rumination	36	20.9
Mental disorder	11	6.4
Mental health of others	6	3.5
**Work**	**65**	**37.8**
High workload	39	22.7
Working conditions	29	16.9
Working atmosphere	9	5.2
Fewer clients	6	3.5
**Restrictions**	**57**	**33.1**
**Global crises**	**46**	**26.7**
War	42	24.4
Pandemic	12	7.0
Climate crisis	6	3.5
**Physical health**	**45**	**26.2**
Somatic complaints	41	23.8
Physical health of others	9	5.2
**Friends and Family**	**28**	**16.3**
Children	25	14.5
Interpersonal problems	14	8.1
**Dissatisfaction with societal development**	**28**	**16.3**
General societal development	19	11
Politics and media reporting	13	7.6
**No or minor negative effects**	**22**	**12.8**
**Finances**	**20**	**11.6**
General worries	13	7.6
Inflation	7	4.1
**Unknown future**	**9**	**5.2**

#### Mental health

The largest category, mentioned by *N*=133 (77.3%) respondents, relates to aspects of mental health and includes six subcategories (Table 2).

Within this category, we saw that 48.8% (*N*=84) clinical psychologists felt most burdened by negative feelings, e.g., unhappiness, fear, tension, or anger, as well as the absence of positive feelings.

*N*=63 (36.6%) wrote about burnout symptoms. Sleep problems, fatigue, and lack of energy were reported particularly frequently, for example, by *respondent 219*, who stated, *“tiredness, falling asleep very quickly on the couch in the evening after the children are in bed, hardly have any energy for things that I could enjoy myself”* when asked about how her burdens currently show themselves.

Another *N*=57 (33.1%) clinical psychologists addressed “excessive demand” as a burden. In their statements, respondents described feelings of stress and problems with time management, such as *“too many tasks at once: very busy day, job, diagnostic findings, everyday life with two children, dog” (respondent 270)*. In this context, respondents also mentioned an increase in negative habits (e.g., screen time, alcohol consumption, procrastination) and decreased positive habits (e.g., regular exercise, social gatherings, healthy eating).

*N*=36 (20.9%) respondents felt burdened by rumination. They wrote about constant worrying and circling of thoughts.

*N*=11 (6.4%) referred to a specific mental health disorder, and *N*=6 (3.5%) were worried about mental health and harmful habits of friends and family.

#### Work

*N*=65 (37.8%) respondents named aspects of work as a burden. This main category comprised 4 subcategories (Table 2).

*N*=39 (22.7%) respondents experienced a high workload as a burden. Respondents were troubled by long working hours, a high number of patient requests, and increased mental disorders among their patients. Respondent 403, who reported an extreme influx to the practice and feeling unable to help enough, wrote: *“We don't know where to send people anymore; all colleagues are overloaded”*. Statements also included direct consequences of this situation on their work, e.g., poor treatment quality.

Further, *N*=29 (16.9%) respondents mentioned burdens due to working conditions. Aspects like an uncertain working situation, poor pay, postponed appointments, or irregular working hours were found to be challenging.

Other *N*=9 (5.2%) respondents described burdens related to the workplace atmosphere, such as interpersonal problems within the team and conflict at the workplace. Moreover, *N*=6 (3.5%) respondents felt burdened by a lack of patients.

#### COVID-19 restrictions

Another area of concern mentioned by *N*=57 (33.1%) respondents relates to COVID-19 restrictions. Statements referred to restrictions such as lockdowns, compulsory vaccination or masks, and their consequences. Limited opportunities for recreational activities and lack of social contact were repeatedly addressed. Respondent 24, displeased by the measures, wrote: *“I miss shaking hands and hugs.”* Contrary to that, the absence and relaxation of restrictions were also perceived negatively, as respondents felt insufficiently protected.

#### Global crises

*N*=46 (26.7%) respondents named worries about three current global crises and their consequences a burden. The three subcategories are summarized in Table 2.

*N*=42 (24.4%) respondents made statements relating to the current Ukraine-Russia conflict.

Further, *N*=12 (7%) addressed the “pandemic” as a burden. Within this category, almost exclusively single-word responses like “pandemic” or “corona” were coded.

Moreover, *N*=6 (3.5%) said they were concerned about the climate crisis.

#### Physical health

*N*=45 (26.2%) respondents reported physical health complaints. *N*=41 (23.8%) mentioned general concerns about their health as well as specific symptoms such as somatic pain, muscle tension, and gastrointestinal problems.

Another *N*=9 (5.2%) were concerned about the physical health and death of close people.

#### Other burdens

*N*=28 (16.3%) respondents reported burdens related to their friends and family. For *N*=25 (14.5%), such burdens referred to issues associated with their children, e.g., children’s progress at school or childcare. *N*=14 (8.1%) described interpersonal problems, which were often related to differing attitudes toward COVID-19.

A further category, named by *N*=28 (16.3%) respondents, concerns dissatisfaction with societal development. *N*=19 (11%) expressed dissatisfaction with the general societal development. Respondent 634, for example, stated: *“The increase in people's inability to hold conversations and lack of tolerance to accept other points of view. The split of the middle class. That people are more incapable regarding their social competencies (interpersonal interaction, empathy).”* For *N*=13 (7.6%) the dissatisfaction was related to politics and media reporting regarding COVID-19 or other topics.

Another area of burden, addressed by *N*=20 (11.6%) respondents, refers to finances. In this context, *N*=13 (7.6%) reported general worries regarding their personal financial situation, and *N*=7 (4.1%) were concerned about inflation.

*N*=9 (5.2%) made vague statements that they were worried about the distant future.

### Resources

Among the questions related to resources (questions 4-5), *N*=150 (87.2%) answered at least one question, and *N*=133 (77.3%) answered both questions.

**Fig. 2:**
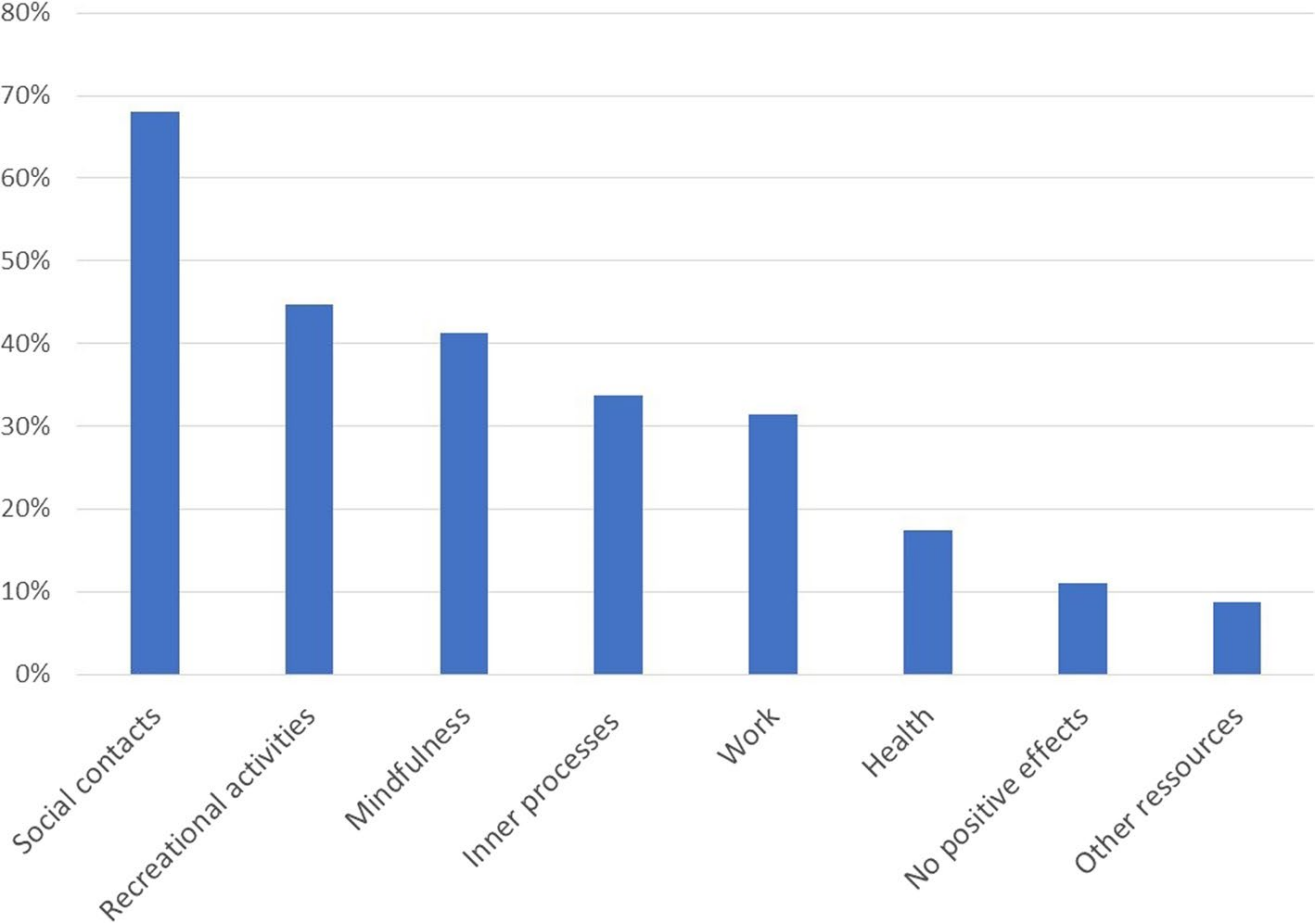
Resources respondents accessed to deal with burdens. The percentages of respondents reporting one or more resources in each of the main categories that resulted from the qualitative content analysis of questions 4-5: (Question 4) What helped you to cope with the adverse effects of the pandemic? And (Question 5) Have there also been positive effects due to the pandemic?

Qualitative content analysis resulted in eight categories and 23 subcategories. Fig. 2 depicts the percentages of the main resource categories. All findings are described in detail in Table 3.

**Table 3.** Category system that emerged from the qualitative content analysis of questions 4-5: (Question 4) What helped you to cope with the adverse effects of the pandemic? And (Question 5) Have there also been positive effects due to the pandemic?

	***N***	**%**
**Social contacts**	**117**	**68.0**
Partners, family and friends	84	48.8
Other social contacts	35	20.3
Colleagues	15	8.7
Fewer social contacts/responsibilities	13	7.6
Pets	9	5.2
**Recreational activities**	**77**	**44.7**
Being outside	38	22.1
Sports	37	21.5
Hobbies	26	15.1
**Mindfulness**	**71**	**41.3**
Slowing down	40	23.3
Prioritizing	29	16.9
Mental techniques and exercises	29	16.9
**Inner Processes**	**58**	**33.7**
Positive attitude	24	14.0
Resilience	23	13.4
Self-reflection	21	12.2
**Work**	**54**	**31.4**
Flexible working conditions	30	17.4
Working	17	9.9
Recognition for psychosocial services	8	4.7
Less work	8	4.7
**Health**	**30**	**17.4**
Focus on hygiene and health	19	11.0
Professional support	11	6.4
**No positive effects**	**19**	**11.0**
**Other resources**	**15**	**8.7**
Structure	7	4.1
Increase in financial resources	5	2.9
Vacations	4	2.3

#### Social contacts

The category “social contacts”, an important resource for 68% (*N*=117) of the respondents, consists of five subcategories (Table 3).

Most respondents (*N*=84; 48.8%) mentioned “Partners, family and friends” as a source of support. Respondents felt it was a resource to spend more time together and have a stronger connection with close family and friends, which they associated with having more time than usual during the pandemic.

Further, *N*=35 (20.3%) respondents mentioned other social contacts and conversations in general as a resource, and *N*=15 (8.7%) referred to colleagues as a social support network.

While most respondents drew on social contacts as a resource, *N*=13 (7.6%) stated that they felt relieved by having fewer social contacts and obligations, e.g., family gatherings or more options for social withdrawal when needed during the pandemic.

Finally, *N*=9 (5.2%) referred to their pets as a resource.

#### Recreational activities

*N*=77 (44.7%) respondents mentioned recreational activities as a resource. The main category comprises three subcategories displayed in Table 3. Across all statements, respondents emphasized that having time for themselves was a positive effect of the pandemic.

*N*=38 (22.1%) respondents enjoyed being outside in nature. This subcategory included going for walks, spending time, or meeting friends outside.

Exercising was perceived as helpful by *N*=37 (21.5%) respondents. Statements within the subcategory “sports” encompass activities such as running, weightlifting, martial arts, mountain climbing, or just investing more time in doing sports.

*N*=26 (15.1%) respondents mentioned that finding new or indulging in existing hobbies, e.g., cooking, baking, making or listening to music, creating art, writing, watching movies, gardening, or reading, was a resource during the pandemic.

#### Mindfulness

Practising mindfulness was a resource for *N*=71 (41.3%) respondents.

The calmness and deceleration of pace in everyday life, summarised in the subcategory “slowing down”, was mentioned by *N*=40 (23.3%) respondents. Respondents indicated there was less pressure to use leisure time productively and that, especially during curfews, they participated less in public life and retreated to the private sphere.

Further, *N*=29 (16.9%) respondents referred to “prioritizing” as a resource. They reported that concentrating on important things, focusing on or changing priorities, focusing on oneself or one’s own life, and finding clarity on what is important were positive effects of the pandemic. Prioritizing could be considered a mental mindfulness technique but differs from these by the passive nature of the formulation of responses. Respondents did not refer to specific practices or techniques but to states of serenity due to a decelerated environment.

*N*=29 (16.9%) respondents referred to a related but distinct subcategory comprising particular exercises, including techniques for mindfulness, relaxation, meditation, breath work, and emotionality. They voiced being actively mindful of small things and living in the moment.

#### Inner processes

Another main category *N*=58 (33.7%) reported as a resource during the pandemic, relates to inner processes. As summarized in Table 3, *N*=24 (14%) respondents mentioned their “positive attitude” as a resource throughout the pandemic. Respondents described focusing on the positive side of things and looking to the future with confidence. For example, respondent 1 reported*: “my generally positive attitude towards things, which actively counteracts when I notice my mood getting bad.”*

*N*=23 (13.4%) respondents described their flexibility and adaptability in dealing with the pandemic as a resource. They named their courage, emotional stamina, confidence, and competence to handle a situation or face fears. We subsumed these statements under the subcategory “resilience”, which can be defined as the process of successfully navigating, adapting to, or managing adversity, stressors or traumatic experiences [41].

*N*=21 (12.2%) respondents mentioned self-reflection as a resource and reported confronting their feelings. For example, respondent 110 described the pandemic as an *“opportunity to become aware of and integrate one’s fears”*. Actively reflecting on the pandemic situation also helped respondents to develop new perspectives in dealing with COVID-19 measures. As respondent 158 expressed, *“I have tried to put the processes and measures in perspective. My attitude is: there are a lot worse things than having to wear masks.”*

#### Work

Work-related changes due to the pandemic were relevant for *N*=54 (31.4%) respondents. Subcategories are displayed in Table 3.

The pandemic entailed working from home and changed working conditions in many places. Positive mentions of this also came from the respondents in our study and were subsumed under the subcategory “flexible working conditions”, which was named by *N*=30 (17.4%) respondents. They described working digitally or from home, which saved time and mental resources.

Further *N*=17 (9.9%) respondents mentioned their work in general as a resource during the pandemic.

*N*=8 (4.7%) commented positively on increased recognition of psychosocial services by political actors, the media, or society in general. Related to this, they observed a greater number of patients and professional inquiries.

At the same time, *N*=8 (4.7%) respondents reported that they noticed a decreased workload, which was also experienced positively. Respondents reported fewer clients, fewer appointments and work commitments, especially during curfews in the first year of the pandemic.

#### Health

A positive effect of the pandemic, mentioned by *N*=30 (17.4%) respondents, was the increased importance of health.

*N*=19 (11%) respondents noticed a stronger drive to maintain physical and mental health. They supported the introduction of protective measures against diseases, including COVID-19.

*N*=11 (6.4%) respondents additionally reported seeking professional support related to their health, such as supervision, psychotherapy, or physiological medical care.

#### Other resources

In addition, *N*=7 (4.1%) respondents mentioned structure, routines, and self-organization in their private and professional environment as a resource throughout the pandemic. An increase in available financial resources was noted in *N*=5 (2.9%) cases due to lower expenses or higher income. Further *N*=4 (2.3%) respondents drew resources from vacations.

## Discussion

The study illuminates the significant challenges faced by clinical psychologists during the pandemic, with mental health-related issues and work-related stressors being prominent concerns. These findings are consistent with previous research highlighting the strain experienced by healthcare workers during the COVID-19 crisis [19, 20, 42]. However, specific findings for mental healthcare professionals demonstrated variations in experiences [21, 24, 32, 36]. The extent and nature of these challenges may vary across countries due to differences in healthcare systems, pandemic management strategies, and cultural factors.

In Austrian clinical psychologists lower rates of clinically relevant mental health problems compared to the general population were observed [35]. Results of the current study suggest heightened awareness of the pandemic’s impact in this specific group of healthcare professionals compared to the general public. While mental health was the most prominent main burden category in clinical psychologists (mentioned by 77.3%), it was less frequently mentioned (10.5% of the total sample) in the general population surveyed at the same time when asked about the current greatest source of problems [43]. However, only 6.4% of the clinical psychologists referred directly to their mental health disorders, which is still lower than the number of clinical psychologists scoring above the cut-offs for clinically relevant mental health problems (12.2% for depression and anxiety, 43% for stress) [35].

Next to mental health issues, work-related themes were frequently mentioned as a burden by the participating clinical psychologists. Clinical psychologists felt burdened by an increased demand for psychological services, which is in line with a recent study on changes in patient numbers of Austrian psychotherapists throughout the pandemic [13]. This study observed that after an initial decline in patient numbers during the first nationwide lockdown in the spring of 2020, patient numbers increased, exceeding pre-pandemic numbers in 2021 and 2022.

The frequent mention of mental health issues and work-related stressors highlights the need for clinical psychologists to foster mental hygiene to provide high-quality services during multilevel crises.

Other burdens mentioned by clinical psychologists reflect the pandemic-related restrictions and the current socio-political and economic situation. However, given that inflation rates experienced a dramatic upsurge in the early months of 2022 [44], the low proportion of clinical psychologists expressing worries about their personal financial situation (7.6%) indicates that this group mainly consists of individuals with financially satisfactory life situations. The preventive role of economic security on mental health is strengthened by multivariable analyses conducted on a representative sample of the Austrian general population surveyed in April 2022, showing that among several sociodemographic factors, household income was one of the variables strongest associated with mental health [11]. The improved financial position of clinical psychologists compared to the general population in Austria is also indicated by the frequent mentions (30.4%) of concerns regarding inflation and finances as the main source of worry among the Austrian general population expressed in a survey in April 2022 [43].

Regarding resources for coping with stress, clinical psychologists were found to rely mainly on positive coping strategies, such as seeking social support, engaging in recreational activities, and practicing mindfulness or positive thinking. These are associated with lower levels of psychological distress [27] and stress symptoms [45] in mental health professionals.

In contrast to the areas of concern, the mentioned resources showed high similarity between clinical psychologists and the general population [43]. In both groups, social contacts had the highest overall score of all resources mentioned. Previous studies support the role of social relationships in mitigating mental health symptoms during the pandemic [46–48]. In a review of 31 studies on the coping behaviors of healthcare workers, Labrague [49] found support from and communication with family, friends and colleagues to be a primary coping mechanism for managing adverse consequences of the COVID-19 pandemic.

Recreation was the second most frequent category mentioned by clinical psychologists and the Austrian general population surveyed in spring 2022 [43]. This category comprised spending time in nature, practicing sports, and finding new or indulging in existing hobbies. The importance of physical activity for mental health has been highlighted in several previous studies [11, 50–52].

Practising mindfulness was the third most vital resource. Respondents were very explicit in naming various mindfulness techniques, possibly due to their professional backgrounds. Previous studies support the potential of mindfulness practice to strengthen resilience and the ability to cope with adversity during crises [53–55].

Focussing on inner processes was reported as an important resource by more than one third of clinical psychologists, with positive thinking, self-confidence, and self-reflection frequently mentioned. The preventive role of a positive attitude is supported by a study conducted on the Austrian general population during the first COVID-19 lockdown, demonstrating that positive thinking was associated with less perceived stress, depression, anxiety, and insomnia [29]. The positive attitude of clinical psychologists is also reflected in the low proportion (11%) of participants stating that the pandemic was not associated with any positive aspect. Waters et al. [56] suggested an interaction between positive emotions and psychological distress. They argued that positive emotions serve to (1) diminish mental health threats, (2) maintain mental health, and (3) enable the individual to use a crisis in a transformative way to develop new perspectives or strategies. Quotes from our study illustrate that clinical psychologists, similar to respondents interviewed by Yang et al. [57], resorted to strategies such as positive refocusing (e.g. turning to the positive things in life) or positive reappraisal (e.g. focusing on what can be learned from the situation).

Work was also mentioned by almost one third of the participating clinical psychologists as an important resource, while it was named by only about 4% of the general population [43]. The frequent mentions of clinical psychologists may have several reasons. For one, the pandemic went along with changes in the clinical psychologists` working conditions, such as increased flexibility due to the possibility of working from home and even treating patients from a distance. Moreover, the high mental health burden in the general population increased the awareness of the importance of mental healthcare services by policymakers, the media, and society in general. It might be possible that self-experienced job-related meaningfulness, a well-known protective factor against job-related distress and associated mental health disorders [33, 58], even increased in clinical psychologists during the pandemic. Research has also shown that supporting others can help people cope better with crises [59, 60]. Supporting their patients through the pandemic may have become a resource for clinical psychologists to better manage themselves.

This study has several limitations. First, the written conduct of the study reduced the possibility of deriving more contextually embedded and coherent information as it would be possible in personal interviews. Second, all questions were asked when less pandemic-related restrictions were in place, which might have caused some recall bias when asked about the burdens and resources experienced during the pandemic. Third, all burdens and resources mentioned are likely also affected by other crises, such as the war in Europe and the associated high inflation rates. Fourth, we have not differentiated how the experiences of different groups differ. For example, it is known from other studies that men and women deal with stress differently [61]. The work context also influences the stresses experienced and offers different ways of dealing with stressful situations. Frenkel et al. [62] showed that healthcare professionals in outpatient facilities experience more stress than those in inpatient contexts. They suggest team commitment and knowledge exchange can help buffer against adverse psychological stress responses. A large proportion of the clinical psychologists we surveyed work in private practice, where there is no guarantee of being part of a team. Winter et al. [13] have shown that the psychotherapists they interviewed wished for more opportunities for intervision, supervision and training to deal with work-related stress. This could also apply to clinical psychologists, so surveying their support needs in further research is necessary. A fifth notable limitation of this study is the low response rate of about 6%, which raises concerns about the representativeness of the sample. The reliance on online data collection may have introduced selection bias, as it is possible that clinical psychologists who chose to participate differed systematically from those who did not. Additionally, the online nature of the survey may have excluded clinical psychologists who do not have access to or are less inclined to participate in online surveys. Therefore, caution should be exercised when extrapolating these findings to the broader population of clinical psychologists. 

## Conclusion

Overall, it seems that clinical psychologists are characterized by a high awareness of mental health-related problems related to the pandemic and the usage of adaptive coping strategies to deal with them. These findings underscore the importance of proactive self-care strategies in maintaining well-being amidst crises.

The evolving work conditions for clinical psychologists, including increased flexibility and heightened awareness of mental healthcare services, highlight the resilience and adaptability of the profession. Future research should explore support needs and interventions for managing work-related stress effectively. Moreover, there is a need to collect more detailed information on the personal experiences of different groups (e.g., vulnerable vs resilient groups or women vs men).

Overall, prioritizing mental health and leveraging available resources are crucial for clinical psychologists to continue providing essential support during challenging times.

## Data Availability

The dataset used and analyzed during the current study is available from the corresponding author upon reasonable request.

## References

[1] Brooks SK, Webster RK, Smith LE, Woodland L, Wessely S, Greenberg N (2020). The psychological impact of quarantine and how to reduce it: rapid review of the evidence. Lancet Br Ed..

[2] Pieh C, Probst T, Budimir S, Humer E (2021). Diminished well-being persists beyond the end of the COVID-19 lockdown. Gen Hosp Psychiatry..

[3] Gallagher MW, Zvolensky MJ, Long LJ, Rogers AH, Garey L (2020). The Impact of Covid-19 Experiences and Associated Stress on Anxiety, Depression, and Functional Impairment in American Adults. Cogn Ther Res..

[4] Fiorillo A, Gorwood P (2020). The consequences of the COVID-19 pandemic on mental health and implications for clinical practice. Eur Psychiatry.

[5] Vienna Center for Electoral Research. Corona-Blog. 2023. Available from: https://covid19.who.int/region/euro/country/athttps://viecer.univie.ac.at/corona-blog/themenuebersicht/

[6] Scherndl G, Mittelstaedt K. Chronologie eines Scheiterns: Impfdrama in vier Phasen. DER STANDARD. 2022 Jun 15; Available from: https://www.derstandard.at/story/2000136871112/impfdrama-in-vier-phasen. Cited 2023 Mar 23.

[7] Pieh C, Budimir S, Probst T (2020). The effect of age, gender, income, work, and physical activity on mental health during coronavirus disease (COVID-19) lockdown in Austria. J Psychosom Res..

[8] Hinz A, Klein AM, Brähler E, Glaesmer H, Luck T, Riedel-Heller SG (2017). Psychometric evaluation of the Generalized Anxiety Disorder Screener GAD-7, based on a large German general population sample. J Affect Disord..

[9] Kocalevent RD, Hinz A, Brähler E (2013). Standardization of the depression screener patient health questionnaire (PHQ-9) in the general population. Gen Hosp Psychiatry..

[10] Pieh C, Budimir S, Humer E, Probst T. Comparing mental health during the COVID-19 lockdown and 6 months after the lockdown in austria: a longitudinal study. Front Psychiatry. 2021;12. Available from: https://www.frontiersin.org/articles/10.3389/fpsyt.2021.625973. Cited 2023 Mar 16. 10.3389/fpsyt.2021.625973 PMC8042148 33859579

[11] Humer E, Schaffler Y, Jesser A, Probst T, Pieh C. Mental health in the Austrian general population during COVID-19: Cross-sectional study on the association with sociodemographic factors. Front Psychiatry . 2022;13. Available from: https://www.frontiersin.org/articles/10.3389/fpsyt.2022.943303. Cited 2023 Mar 16. 10.3389/fpsyt.2022.943303 PMC9729349 36506423

[12] Humer E, Haid B, Schimböck W, Reisinger A, Gasser M, Eichberger-Heckmann H (2021). Provision of Psychotherapy One Year after the Beginning of the COVID-19 Pandemic in Austria. Int J Environ Res Public Health..

[13] Winter S, Jesser A, Probst T, Schaffler Y, Kisler IM, Haid B (2023). How the COVID-19 Pandemic Affects the Provision of Psychotherapy: Results from Three Online Surveys on Austrian Psychotherapists. Int J Environ Res Public Health..

[14] Heidegger KE. European Association for Psychotherapy. 2017. Psychotherapy in Austria. Available from: https://www.europsyche.org/situation-of-psychotherapy-in-various-countries/austria/. Cited 2020 May 25.

[15] Bundesgesetz über die Führung der Bezeichnung „Psychologin“ oder „Psychologe“ und über die Ausübung der Gesundheitspsychologie und der Klinischen Psychologie (Psychologengesetz 2013). BGBl. I Nr. 182/2013 Aug 6, 2013. Available from: https://www.ris.bka.gv.at/GeltendeFassung.wxe?Abfrage=Bundesnormen&Gesetzesnummer=20008552.

[16] Summers EMA, Morris RC, Bhutani GE (2020). A measure to assess the workplace well-being of psychological practitioners. Clin Psychol Psychother..

[17] Aafjes-van Doorn K, Békés V, Prout TA, Hoffman L (2020). Psychotherapists’ vicarious traumatization during the COVID-19 pandemic. Psychol Trauma Theory Res Pract Policy..

[18] Bohlken J, Schömig F, Lemke MR, Pumberger M, Riedel-Heller SG (2020). COVID-19-Pandemie: Belastungen des medizinischen Personals. Psychiatr Prax..

[19] da Silva Neto RM, Benjamim CJR, de Medeiros Carvalho PM, Neto MLR (2021). Psychological effects caused by the COVID-19 pandemic in health professionals: A systematic review with meta-analysis. Prog Neuropsychopharmacol Biol Psychiatry..

[20] Danet AD (2021). Psychological impact of COVID-19 pandemic in Western frontline healthcare professionals. A systematic review. Med Clínica Engl Ed.

[21] Probst T, Humer E, Stippl P, Pieh C. Being a Psychotherapist in Times of the novel coronavirus disease: stress-level, job anxiety, and fear of coronavirus disease infection in more than 1,500 psychotherapists in Austria. Front Psychol . 2020 ;11. Available from: https://www.frontiersin.org/articles/10.3389/fpsyg.2020.559100. Cited 2023 Mar 16. 10.3389/fpsyg.2020.559100 PMC7550677 33132965

[22] Cerasa A, Craig F, Foti F, Palermo L, Costabile A (2022). The impact of COVID-19 on psychologists’ practice: An Italian experience. J Affect Disord Rep..

[23] McDonnell D, Vasiliou VS, Lonergan E, Moore P (2022). Psychologists’ Experiences Who Managed Waitlists in Mental-Health Services During the COVID-19 Lockdown. Eur J Psychol Open..

[24] Rosen CS, Kaplan AN, Nelson DB, La Bash H, Chard KM, Eftekhari A (2023). Implementation context and burnout among Department of Veterans Affairs psychotherapists prior to and during the COVID-19 pandemic. J Affect Disord..

[25] Litam SDA, Ausloos CD, Harrichand JJS (2021). Stress and Resilience Among Professional Counselors During the COVID-19 Pandemic. J Couns Dev..

[26] Schwartzkopff L, Schüller J, Müller-Engelmann M (2022). Burn-On Instead of Burn-Out: Self-Care and Functional Coping Strategies Protect Psychotherapists from Psychological Stress During the Corona Pandemic. Psychother Psychosom Med Psychol..

[27] Babore A, Lombardi L, Viceconti ML, Pignataro S, Marino V, Crudele M (2020). Psychological effects of the COVID-2019 pandemic: Perceived stress and coping strategies among healthcare professionals. Psychiatry Res..

[28] Flesia L, Monaro M, Mazza C, Fietta V, Colicino E, Segatto B (2020). Predicting Perceived Stress Related to the Covid-19 Outbreak through Stable Psychological Traits and Machine Learning Models. J Clin Med..

[29] Budimir S, Probst T, Pieh C (2021). Coping strategies and mental health during COVID-19 lockdown. J Ment Health..

[30] Tavel P, Trnka R, Furstova J, Kascakova N, Kuska M, Meier Z (2022). Dispositional resilience predicted the perceived stress experienced by psychotherapists during the COVID-19 outbreak. Psychol Serv..

[31] Schaffler Y, Kaltschik S, Probst T, Jesser A, Pieh C, Humer E. Mental health in Austrian psychotherapists during the COVID-19 pandemic. Front Public Health. 2022;10. Available from: https://www.frontiersin.org/articles/10.3389/fpubh.2022.1011539. Cited 2023 Mar 16. 10.3389/fpubh.2022.1011539 PMC9679414 36424964

[32] Humer E, Pieh C, Probst T, Kisler IM, Schimböck W, Schadenhofer P (2021). Telephone Emergency Service 142 (TelefonSeelsorge) during the COVID-19 Pandemic: Cross-Sectional Survey among Counselors in Austria. Int J Environ Res Public Health..

[33] Humer E, Pieh C, Kisler IM, Schimböck W, Schadenhofer P (2022). A Longitudinal Study on Mental Well-Being, Perceived Stress Level and Job-Related Meaningfulness of Austrian Telephone Emergency Service Counselors during the COVID-19 Pandemic. Int J Environ Res Public Health..

[34] Tay S, Alcock K, Scior K (2018). Mental health problems among clinical psychologists: Stigma and its impact on disclosure and help-seeking. J Clin Psychol..

[35] Humer E, Pammer B, Schaffler Y, Kothgassner OD, Felnhofer A, Jesser A, et al. Comparison of mental health indicators in clinical psychologists with the general population during the COVID-19 pandemic. Sci Rep. 2023;13(1):5050. 10.1038/s41598-023-32316-x. 10.1038/s41598-023-32316-x PMC10043835 36977787

[36] Campos JADB, Martins BG, Campos LA, de Fátima Valadão-Dias F, Marôco J (2021). Symptoms related to mental disorder in healthcare workers during the COVID-19 pandemic in Brazil. Int Arch Occup Environ Health..

[37] Harris PA, Taylor R, Minor BL, Elliott V, Fernandez M, O’Neal L (2019). The REDCap consortium: Building an international community of software platform partners. J Biomed Inform..

[38] Schaffler Y, Bauer M, Schein B, Jesser A, Probst T, Pieh C (2023). Understanding pandemic resilience: a mixed-methods exploration of burdens, resources, and determinants of good or poor well-being among Austrian psychotherapists. Front Public Health..

[39] Hsieh HF, Shannon SE (2005). Three Approaches to Qualitative Content Analysis. Qual Health Res.

[40] Atlas.ti. ATLAS.ti: The Qualitative Data Analysis & Research Software. 2018. Available from: https://atlasti.com/de/. Cited 2020 Oct 15.

[41] Windle G (2011). What is resilience? A review and concept analysis. Rev Clin Gerontol..

[42] Bohlken J, Schömig F, Lemke MR, Pumberger M, Riedel-Heller SG (2020). COVID-19-Pandemie: Belastungen des medizinischen Personals: Ein kurzer aktueller Review. Psychiatr Prax..

[43] Gächter A, Zauner B, Haider K, Schaffler Y, Probst T, Pieh C (2023). Areas of Concern and Support among the Austrian General Population: A Qualitative Content Analytic Mapping of the Shift between Winter 2020/21 and Spring 2022. Healthcare..

[44] Fessler P, Fritzer F, Salish M. Who pays the price when prices rise?. Monetary Policy & the Economy. Austrian Central Bank. (Q4/22-Q1/):67–84.

[45] Nie A, Su X, Zhang S, Guan W, Li J (2020). Psychological impact of COVID-19 outbreak on frontline nurses: A cross-sectional survey study. J Clin Nurs..

[46] McMahon G, Douglas A, Casey K, Ahern E (2022). Disruption to well-being activities and depressive symptoms during the COVID-19 pandemic: The mediational role of social connectedness and rumination. J Affect Disord..

[47] Mariani R, Renzi A, Di Trani M, Trabucchi G, Danskin K, Tambelli R. The impact of coping strategies and perceived family support on depressive and anxious symptomatology during the coronavirus pandemic (COVID-19) lockdown. Front Psychiatry. 2020;11. Available from: https://www.frontiersin.org/articles/10.3389/fpsyt.2020.587724. Cited 2023 Mar 16. 10.3389/fpsyt.2020.587724 PMC7691226 33281647

[48] Pieh C, Probst T, Budimir S, Humer E (2021). Associations between Relationship Quality and Mental Health during COVID-19 in the United Kingdom. Int J Environ Res Public Health..

[49] Labrague LJ (2021). Psychological resilience, coping behaviours and social support among health care workers during the COVID-19 pandemic: A systematic review of quantitative studies. J Nurs Manag..

[50] Kandola A, Ashdown-Franks G, Hendrikse J, Sabiston CM, Stubbs B (2019). Physical activity and depression: Towards understanding the antidepressant mechanisms of physical activity. Neurosci Biobehav Rev..

[51] Pearce M, Garcia L, Abbas A, Strain T, Schuch FB, Golubic R (2022). Association Between Physical Activity and Risk of Depression: A Systematic Review and Meta-analysis. JAMA Psychiatry..

[52] Schaffler Y, Probst T, Pieh C, Haid B, Humer E (2024). Prevalence of mental health symptoms and potential risk factors among Austrian psychotherapists. Sci Rep..

[53] Zenani NE, Gause G, Sehularo L (2022). Strategies to enhance resilience to cope with workplace adversities post-COVID-19 among ICU nurses. Curationis..

[54] Antonini Philippe R, Schwab L, Biasutti M. Effects of physical activity and mindfulness on resilience and depression during the first wave of cOVID-19 pandemic. Front Psychol. 2021;12. Available from: https://www.frontiersin.org/articles/10.3389/fpsyg.2021.700742. Cited 2023 Mar 16. 10.3389/fpsyg.2021.700742 PMC8360111 34393936

[55] Kunzler AM, Chmitorz A, Röthke N, Staginnus M, Schäfer SK, Stoffers-Winterling J (2022). Interventions to foster resilience in nursing staff: A systematic review and meta-analyses of pre-pandemic evidence. Int J Nurs Stud..

[56] Waters L, Algoe SB, Dutton J, Emmons R, Fredrickson BL, Heaphy E (2022). Positive psychology in a pandemic: buffering, bolstering, and building mental health. J Posit Psychol..

[57] Yang D, Tu CC, Dai X (2020). The effect of the 2019 novel coronavirus pandemic on college students in Wuhan. Psychol Trauma Theory Res Pract Policy..

[58] Schadenhofer P, Kundi M, Abrahamian H, Blasche G, Stummer H, Kautzky-Willer A (2018). Job-related meaningfulness moderates the association between over-commitment and emotional exhaustion in nurses. J Nurs Manag..

[59] Ooi L, Paul E, Burton A, Fancourt D, McKinlay AR (2023). A qualitative study of positive psychological experiences and helpful coping behaviours among young people and older adults in the UK during the COVID-19 pandemic. PloS One..

[60] Lau JTF, Yang X, Tsui HY, Pang E, Wing YK (2006). Positive mental health-related impacts of the SARS epidemic on the general public in Hong Kong and their associations with other negative impacts. J Infect..

[61] Tamres LK, Janicki D, Helgeson VS (2002). Sex Differences in Coping Behavior: A Meta-Analytic Review and an Examination of Relative Coping. Personal Soc Psychol Rev..

[62] Frenkel MO, Pollak KM, Schilling O, Voigt L, Fritzsching B, Wrzus C (2022). Stressors faced by healthcare professionals and coping strategies during the early stage of the COVID-19 pandemic in Germany. PloS One..

